# 气相色谱-质谱法测定大鼠肝脏中的39种脂肪酸

**DOI:** 10.3724/SP.J.1123.2022.09014

**Published:** 2023-05-08

**Authors:** Yingxia WU, Yan MU, Peishan LIU, Yitian ZHANG, Yingxuan ZENG, Zhifeng ZHOU

**Affiliations:** 南方医科大学公共卫生学院卫生检验检疫系,广东省热带病研究重点实验室,广东广州510515; Department of Hygiene Inspection and Quarantine Science, School of Public Health, Southern Medical University, Guangdong Provincial Key Laboratory of Tropical Disease Research, Guangzhou 510515, China

**Keywords:** 气相色谱-质谱, 脂肪酸, 肝脏, gas chromatography-mass spectrometry (GC-MS), fatty acids, liver

## Abstract

肝脏是脂肪酸代谢的主要场所,研究肝脏中脂肪酸组成和含量的变化可以监测机体的健康状态。现有的检测方法存在样本消耗量大、检测出的脂肪酸种类少等缺点。通过比较不同提取方法和衍生化条件,建立了仅用1.1 mg肝脏组织即可测定39种脂肪酸含量的气相色谱-质谱方法。肝脏组织经研磨匀浆,以氯仿-甲醇(1∶2, v/v)提取总脂肪酸后,氮气吹干,加入含5%硫酸的甲醇溶液,100 ℃反应90 min使脂肪酸甲酯化,经SP-2560色谱柱分离后进质谱仪分析。结果表明,39种脂肪酸甲酯在各自的浓度范围内线性关系良好,相关系数(*R*^2^)大于0.9940。将各脂肪酸甲酯的检出限(LOD)和定量限(LOQ)换算至肝脏中的含量,分别为2~272 ng/mg和7~906 ng/mg。以十三烷酸和二十三烷酸考察方法的加标回收率及精密度,在3个水平下的回收率为82.4%~101.0%,日内相对标准偏差(*n*=5)为3.2%~12.0%,日间相对标准偏差(*n*=3)为5.4%~13.4%。动物暴露于经典的持久性有机污染物全氟辛烷磺酸(PFOS)后,体内脂肪酸的代谢可能会出现异常。将该方法应用于健康和经口暴露PFOS的雄性SD大鼠肝脏中脂肪酸含量的检测,从两组大鼠中均检出了26种脂肪酸,其中C15∶0、C18∶3n6和C18∶1n9t为其他方法未检出的脂肪酸,C18∶1n9t和C18∶1n9c为其他方法未能分离的顺反式同分异构体脂肪酸。该方法操作简便,试剂及样品用量少,灵敏度高,能检测出种类更多的脂肪酸,并能有效分离顺反式同分异构体脂肪酸C18∶1n9t、C18∶1n9c和C18∶2n6t、C18∶2n6c,适用于临床诊断和动物研究中肝脏脂肪酸的组成和含量检测。

## Abstract (en)

Fatty acids not only form phospholipids that contribute to the formation of cell membranes but also participate in many metabolic activities, such as energy storage and cell signal transduction. The liver plays a key role in the synthesis and metabolism of fatty acids. The composition and contents of fatty acids in the liver are closely related to body health. Most fatty acid-detection methods require a large sample size and can detect only a small number of fatty acids. Therefore, a sensitive and efficient method to determine fatty acids in the liver is urgently required. Herein, a method based on gas chromatography-mass spectrometry (GC-MS) was established for the simultaneous determination of 39 fatty acids in 1.1 mg of liver tissue. Different extraction methods and derivatization conditions were compared to develop an optimal sample-treatment method. The performance of two different columns in separating the target fatty acids were also compared. A total of 10 mg of liver was added to 450 μL of normal saline and ground at -35 ℃ to obtain a homogenate. Next, 50 μL of the homogenate (equivalent to 1.1 mg of liver) was added with 750 μL of chloroform-methanol (1∶2, v/v) to extract total fatty acids. The fatty acid extracts were dried under nitrogen, and then derivatized at 100 ℃ for 90 min after being added with methanol containing 5% sulfuric acid. The fatty acid methyl esters were extracted with hexane and then separated on an SP-2560 capillary column (100 m×0.25 mm×0.2 μm; Supelco, USA) via GC-MS. The results revealed that all 39 fatty acid methyl esters detected had good linearities in the certain mass concentration ranges with correlation coefficients (*R*^2^) greater than 0.9940. The limits of detection (LOD) and quantification (LOQ) of these methyl esters in the liver were 2-272 ng/mg and 7-906 ng/mg, respectively. The accuracy and precision of the method were evaluated by spiking the liver homogenate with tridecylic acid and eicosanoic acid at low (0.09 μg/mg), moderate (0.90 μg/mg), and high (5.40 μg/mg) concentration levels. The recoveries ranged from 82.4% to 101.0% with an intraday relative standard deviations (RSDs) (*n*=5) of 3.2%-12.0% and interday RSDs (*n*=3) of 5.4%-13.4%. The method was successfully applied to detect fatty acids in the livers of four healthy male Sprague-Dawley (SD) rats and four male SD rats with abnormal liver function induced by perfluorooctane sulfonate (PFOS). PFOS is a persistent organic pollutant. Twenty-six fatty acids were detected in the livers of both groups. Among the fatty acids investigated, pentadecanoic acid (C15∶0), *γ*-linolenic acid (C18∶3n6), and elaidic acid (C18∶1n9t) cannot be detected by the methods reported in the literature. By contrast, the method developed in this study could separate the isomers of oleic acid (elaidic acid, C18∶1n9t; oleic acid, C18∶1n9c) and linolenic acid (linolelaidic acid, C18∶2n6t; linoleic acid, C18∶2n6c). In conclusion, the developed method is simple and can detect a large number of fatty acids using small sample amounts and few reagents. More importantly, it could successfully separate fatty acid isomers. These findings indicate that the developed method is suitable for the detection of fatty acid composition and contents in the liver in clinical and experimental research.

脂肪酸是细胞膜的重要组成成分,参与人体能量储存、细胞信号转导等代谢活动^[1]^。肝脏是体内脂肪酸合成和代谢的主要场所^[2]^。肝脏中脂肪酸的种类及含量不仅可以反映机体的健康现状,其变化还会影响机体疾病的转化。非酒精性脂肪肝病(NAFLD)患者肝脏中十八烷酸与十六烷酸的含量比值与肝脏脂肪变性程度存在负相关关系,该含量比值越低,伴随着越严重的肝脏脂肪变性^[3]^。Matsuzaka等^[4]^的研究显示,肝脏中与脂肪酸延长相关的Elovl6酶被抑制后,棕榈酸盐转化为硬脂酸盐会减少,有利于改善胰岛素抵抗和降低心血管风险。肝脏中脂肪酸的检测对于开展更多疾病机理和预防的研究十分重要。

气相色谱法(GC)和气相色谱-质谱法(GC-MS)是组织中脂肪酸检测最常用的方法,但现有的方法大多用于羊肉^[5]^、蟹肉^[6]^等食品类组织样品的检测。肝脏中脂肪酸的提取方法主要有氯仿-甲醇(1∶2, v/v)提取法^[7]^、氢氧化钾-甲醇提取法^[8]^等。提取得到的脂肪酸需进一步甲酯化后方可用GC或GC-MS检测。脂肪酸甲酯化方法有酸催化法^[6⇓⇓-9]^(如硫酸-甲醇溶液为衍生化试剂)和碱催化法^[10]^(如氢氧化钾-甲醇溶液为衍生化试剂)等。现有的方法通常需要0.1~5 g组织样本,仅能检测13~24种脂肪酸^[3,5,6,9]^,且未能分离其中的同分异构体脂肪酸,难以满足临床诊断和实验研究的需要。本研究通过比较和优化脂肪酸提取方法、衍生化条件等,建立了使用更少肝脏样本量即能检测更多脂肪酸(特别是同分异构体脂肪酸)的分析方法。

全氟辛烷磺酸(PFOS)是一种持久性有机污染物,可经过食物、水源、土壤等途径进入体内,从而引起肝脏、神经、内分泌等毒性^[10]^。PFOS暴露可影响肝脏中脂肪酸的代谢^[11]^。本研究将建立的检测方法应用于健康SD大鼠和经口暴露PFOS的SD大鼠(PFOS暴露大鼠)的肝脏中脂肪酸的检测。与已有的方法^[3,9]^相比,本方法能从SD大鼠中检测更多的脂肪酸,同时也可分离文献中未能分离的同分异构体脂肪酸。

## 1 实验部分

### 1.1 仪器、试剂与材料

QP2010 Plus GC-MS(日本Shimadzu公司),配AOC-20i+s自动进样器;CT15RT高速冷冻离心机(上海天美科学仪器有限公司);冷冻研磨仪(广州露卡测序仪器有限公司); Milli-Q纯水仪(美国Millipore公司)。

所有脂肪酸甲酯和脂肪酸标准品均购自美国NU-CHEK公司,其中顺-13,16,19-二十二碳三烯酸甲酯(C22∶3)、顺-7,10,13,16-二十二碳四烯酸甲酯(C22∶4n6)、顺4,7,10,13,16-二十二碳五烯酸甲酯(C22∶5)、十九烷酸甲酯(C19∶0,内标)、十三烷酸(质控)及二十三烷酸(质控)的纯度均大于99%。36种脂肪酸甲酯混合标准品中的棕榈酸(C16∶0)的质量分数为5.26%,其余35种脂肪酸甲酯的质量分数均为2.63%。甲醇(色谱纯,美国Merck公司);正己烷(色谱纯,德国CNW公司);硫酸、氯仿(分析纯,广州化学试剂厂)。

### 1.2 标准溶液的配制

母液A:称取36种脂肪酸甲酯混合标准品200.0 mg,用二氯甲烷-正己烷(1∶2, v/v)(下文简称:稀释溶剂)定容至10 mL。母液B:分别称取C22∶3、C22∶4n6、C22∶5标准品各10.0 mg,混合后用稀释溶剂溶解并定容至10 mL。内标(IS)储备液:称取C19∶0标准品10.0 mg,用稀释溶剂溶解并定容至10 mL。质控储备液:称取十三烷酸和二十三烷酸标准品各10.0 mg,用正己烷-甲醇(1∶4, v/v)溶解并定容至10 mL。

用稀释溶剂将IS储备液稀释成50 μg/mL的IS标准工作液。取适量母液A与B混合后用稀释溶剂依次稀释,配制成含39种脂肪酸甲酯的系列标准工作液,取不同浓度的标准溶液各80 μL和IS标准工作液20 μL混合后由GC-MS测定。用正己烷-甲醇(1∶4, v/v)将质控储备液稀释为10、100、600 μg/mL的质控标准溶液。

### 1.3 实验方法

#### 1.3.1 动物实验

8~10周的雄性SD大鼠8只,购自南方医科大学动物中心,于南方医科大学SPF动物实验部饲养(动物实验伦理审查号:L2018202)。大鼠适应性喂养1周后,随机分为对照组(健康SD大鼠)和PFOS暴露组,每组4只。灌胃剂量:PFOS暴露组为PFOS 5 mg/(kg·d),健康SD大鼠组为0.9%生理盐水5 mL/(kg·d)。暴露32天后,对动物以麻醉后放血的方式处以安乐死,处死后迅速剥离肝脏并称重。取部分肝脏进行病理切片,剩余的肝脏经液氮迅速冷冻后转入-80 ℃冰箱中保存,待测。

#### 1.3.2 组织研磨

每10.0 mg肝脏组织加入生理盐水450 μL,混匀后放入钢珠,在-35 ℃的研磨仪中,以70 Hz的振动频率研磨1 min得到肝脏匀浆。

#### 1.3.3 总脂肪酸的提取

取肝脏匀浆50 μL(相当于肝脏1.1 mg),依据刘佩珊等^[7]^报道的方法提取总脂肪酸,即加入纯水250 μL、氯仿-甲醇(1∶2, v/v)750 μL和氯仿250 μL,涡旋1.5 min,于4 ℃以13 000 r/min离心5 min,转移下层有机相至玻璃管中;向水相中加入氯仿250 μL,重复提取一次,合并两次提取液,氮气吹干,得干燥脂肪酸提取物。

#### 1.3.4 脂肪酸甲酯化

向干燥脂肪酸提取物中加入含5%硫酸的甲醇溶液2 mL,密封后混匀,于100 ℃反应90 min。待反应瓶冷却至室温后,加入超纯水2 mL、正己烷2 mL,混匀后,以13 000 r/min离心5 min,取有机层,氮气吹干,加入正己烷80 μL和IS标准工作液20 μL,混匀,待GC-MS分析。

#### 1.3.5 GC-MS分析条件

色谱条件:SP-2560色谱柱(100 m×0.25 mm×0.2 μm,美国Supelco公司);升温程序:初始温度100 ℃,保持5 min,以4 ℃/min升至240 ℃,保持30 min;进样口温度:230 ℃;分流比10∶1;载气:氦气(纯度大于99.999%),流量:1.0 mL/min;进样量:1 μL。

质谱条件:电子轰击电离(EI)源;离子源温度:250 ℃;接口温度:240 ℃;溶剂延迟4 min;扫描模式:全扫描;质谱扫描范围:*m/z* 33~450。

## 2 结果与讨论

### 2.1 色谱柱的选择

由图1可见,包括C18∶1n9t、C18∶1n9c等同分异构体在内的所有脂肪酸甲酯在SP-2560色谱柱(100 m×0.25 mm×0.2 μm,美国Supelco公司)和DB-23色谱柱(60 m×0.25 mm×0.15 μm,美国Agilent公司)上均能得到较好的分离。

**图1:**
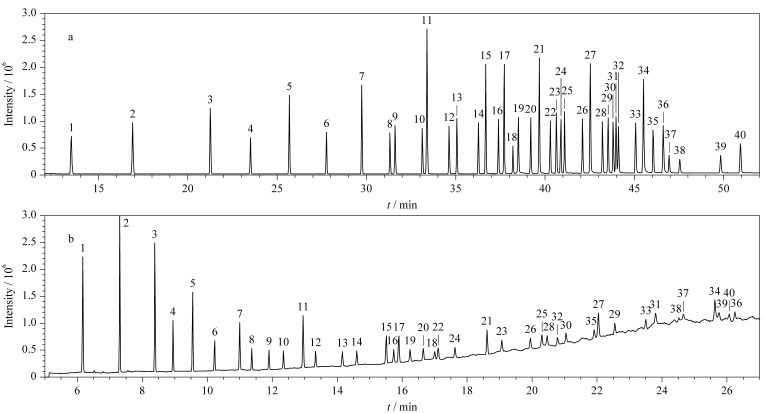
39种脂肪酸甲酯混合标准溶液和内标在(a) SP-2560和(b) DB-23色谱柱上的总离子流色谱图

虽然DB-23色谱柱的分离时间更短,但对于碳链较长的脂肪酸甲酯灵敏度很差,响应仅为在SP-2560色谱柱上分离时的1/2甚至更低。而SP-2560色谱柱虽然因为柱长较长,分离时间比DB-23色谱柱长,但其基线稳定,不同脂肪酸的响应都较高,不会随着碳链数的增长灵敏度下降。为了能检测更多含量较低但有重要生理意义的长链脂肪酸,如顺4,7,10,13,16-二十二碳五烯酸(DPA)^[12]^,本研究选择SP-2560色谱柱进行后续实验。

### 2.2 脂肪酸提取方法的选择

本研究以含5%硫酸的甲醇溶液为衍生化试剂,比较“一步法”和“两步法”的提取效率。“一步法”即使用氯仿-甲醇(1∶2, v/v)进行提取,详细方法见1.3.3节。“两步法”则是使用含0.4 mol/L氢氧化钾的甲醇溶液作为提取试剂^[8]^,两步提取得到的脂肪酸相加即得总脂肪酸含量。

由图2可知,“一步法”提取总脂肪酸、n-6脂肪酸、饱和脂肪酸、单不饱和脂肪酸和多不饱和脂肪酸的效率均显著高于“两步法”(*P*<0.05),其中提取得到的总脂肪酸含量甚至比“两步法”高30%以上。与“两步法”相比,“一步法”提取步骤更简单,使用的试剂更少,且提取的脂肪酸效率更高。为此,本研究选用“一步法”作为总脂肪酸的提取方法。

**图2:**
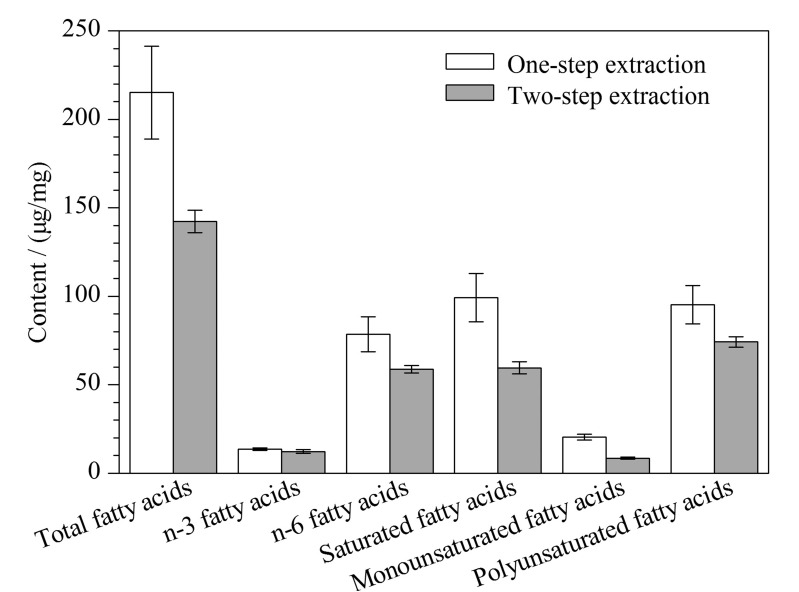
提取方法对肝脏中脂肪酸含量的影响(*n*=3)

### 2.3 衍生化方法的选择和优化

使用“一步法”提取总脂肪酸,比较3种不同衍生化方法的甲酯化效率:方法A,含1 mol/L氢氧化钾的甲醇溶液30 ℃下衍生化9 min^[13]^;方法B,乙酰氯-甲醇(1∶8, v/v)溶液90 ℃下衍生化90 min^[7]^;方法C, 含5%硫酸的甲醇溶液80 ℃下衍生化90 min^[14]^。使用方法A进行衍生化,只能获得15种脂肪酸甲酯目标物;使用方法B和方法C进行衍生化时,均能获得26种脂肪酸甲酯,但是方法C能得到的n-3脂肪酸、n-6脂肪酸、饱和脂肪酸、多不饱和脂肪酸和总脂肪酸含量相对更高(见图3)。因此,对方法C的衍生化试剂用量、温度和时间等参数进一步优化。

**图3:**
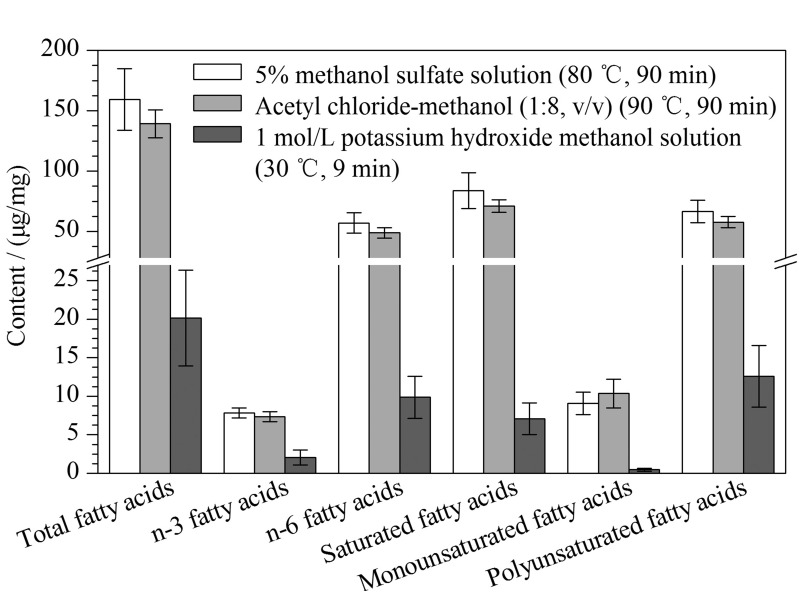
不同衍生化方法对肝脏中脂肪酸含量的影响(*n*=3)

进一步考察了不同体积分数(1%、2.5%、5%、10%)的硫酸甲醇溶液、不同衍生化温度(40、60、80、90、100 ℃)和衍生化时间(30、60、90、120 min)对脂肪酸甲酯化效果的影响。结果显示,肝脏脂肪酸提取物以含5%硫酸的甲醇溶液为衍生化试剂,在100 ℃下甲酯化90 min时,得到的目标物含量最高,甲酯化效果最好,详见图4。

**图4:**
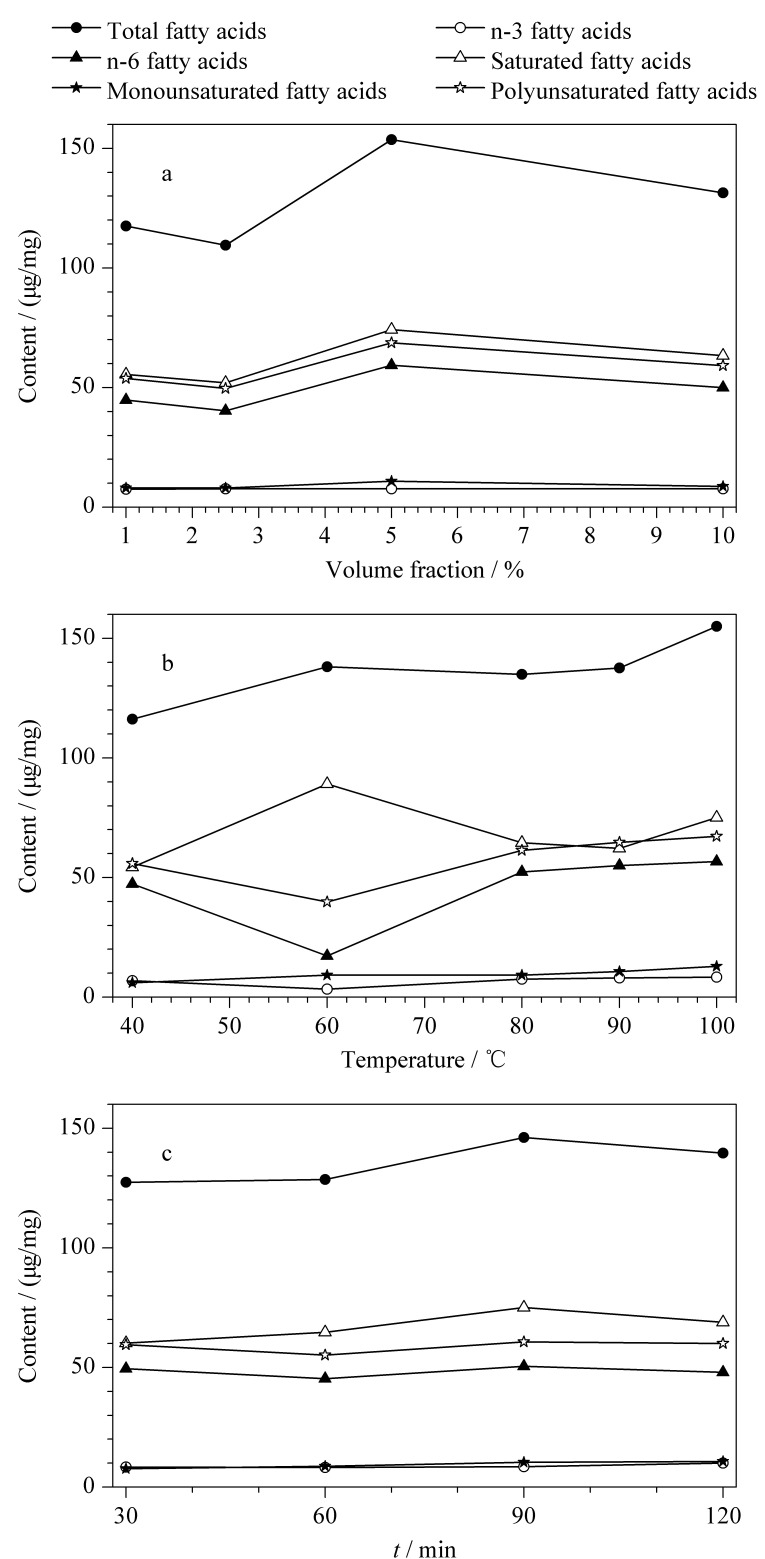
(a)甲醇中硫酸的体积分数、(b)衍生化温度和(c)衍生化时间对肝脏中脂肪酸含量的影响(*n*=3)

### 2.4 方法学考察

#### 2.4.1 线性范围、检出限及定量限

在最优色谱条件下,检测不同浓度的39种脂肪酸甲酯和内标混合标准工作溶液,以脂肪酸甲酯与内标的色谱峰面积比(*y*)为纵坐标,对应的质量浓度比(*x*)为横坐标绘制标准曲线。各脂肪酸甲酯在对应的线性范围内线性相关系数(*R*^2^)为0.9940~1.0000。

以3倍信噪比确定脂肪酸甲酯的检出限(LOD), 10倍信噪比确定定量限(LOQ),将各脂肪酸甲酯的LOD和LOQ换算至肝脏中的含量,分别为2~272 ng/mg和7~906 ng/mg(见表1)。

**表1:** 39种脂肪酸甲酯的线性范围、相关系数、检出限和定量限

Compound	Linear range/ (μg/mL)		*R*^2^	LOD/ (μg/mg)	LOQ/ (μg/mg)	Compound	Linear range/ (μg/mL)		*R*^2^	LOD/ (μg/mg)	LOQ/ (μg/mg)
C6∶0	0.115-	28.680	0.9997	0.004	0.013	C18∶3n6	0.115-	28.680	0.9983	0.074	0.246
C8∶0	0.115-	28.680	0.9997	0.004	0.012	C20∶1	0.115-	17.208	0.9995	0.013	0.043
C10∶0	0.115-	28.680	0.9982	0.004	0.014	C18∶3n3	0.574-	57.360	0.9981	0.075	0.249
C11∶0	0.115-	28.680	0.9940	0.005	0.018	C21∶0	0.115-	17.208	0.9976	0.003	0.011
C12∶0	0.115-	17.208	0.9971	0.008	0.026	C20∶2	1.147-	57.360	0.9996	0.012	0.040
C13∶0	0.115-	286.801	0.9977	0.007	0.022	C22∶0	0.115-	28.680	0.9945	0.002	0.007
C14∶0	0.115-	28.680	0.9998	0.009	0.030	C20∶3n6	1.147-	172.081	0.9997	0.015	0.050
C14∶1	0.115-	28.680	0.9967	0.034	0.113	C22∶1n9	0.115-	28.680	0.9990	0.008	0.025
C15∶0	0.115-	28.680	0.9996	0.014	0.047	C20∶3n3	0.574-	28.680	0.9978	0.147	0.492
C15∶1	0.115-	28.680	0.9985	0.021	0.068	C23∶0	0.057-	286.801	0.9970	0.008	0.025
C16∶0	11.472-	803.044	0.9989	0.003	0.011	C20∶4n6	5.736-	401.522	0.9961	0.006	0.021
C16∶1	0.574-	28.680	0.9952	0.125	0.416	C22∶2	0.115-	28.680	0.9968	0.007	0.023
C17∶0	1.147-	172.081	1.0000	0.015	0.051	C24∶0	0.115-	28.680	0.9986	0.005	0.016
C17∶1	0.574-	28.680	0.9978	0.107	0.356	C20∶5	0.115-	28.680	0.9988	0.019	0.062
C18∶0	5.736-	401.522	0.9995	0.002	0.007	C24∶1	0.574-	57.360	0.9974	0.019	0.063
C18∶1n9t	0.574-	57.360	0.9985	0.137	0.457	C22∶3	0.402-	60.300	0.9999	0.015	0.049
C18∶1n9c	5.736-	286.802	0.9973	0.021	0.069	C22∶4n6	0.382-	57.300	0.9986	0.014	0.045
C18∶2n6t	0.574-	28.680	0.9961	0.272	0.906	C22∶5	0.438-	65.700	0.9988	0.010	0.033
C18∶2n6c	5.736-	401.522	0.9967	0.008	0.027	C22∶6n3	2.868-	286.801	0.9998	0.003	0.010
C20∶0	0.115-	17.208	0.9991	0.002	0.007						

#### 2.4.2 加标回收率及精密度

以十三烷酸和二十三烷酸为研究目标,将3个水平的质控标准溶液添加至不含十三烷酸和二十三烷酸的大鼠肝脏匀浆液中,使得肝脏组织中目标物的含量分别为0.09、0.90、5.40 μg/mg。每个含量水平平行实验5次,并连续测定3天,分别计算日内和日间的相对标准偏差(RSD),考察方法的加标回收率和精密度(以RSD表示)。十三烷酸和二十三烷酸的加标回收率为82.4%~101.0%,日内RSD(*n*=5)为3.2%~12.0%,日间RSD(*n*=3)为5.4%~13.4%(见表2)。结果表明,本方法准确可靠,精密度良好,可以满足肝脏中脂肪酸的定量分析。

**表2:** 添加至肝脏中的十三烷酸和二十三烷酸的加标回收率、日内和日间相对标准偏差

Compound	Spiked/ (μg/mg)	Recovery (*n*=5)/%	Intra-day RSD (*n*=5)/%	Inter-day RSD (*n*=3)/%
Tridecanoic	0.09	85.9	9.9	8.2
acid	0.90	101.0	5.3	5.4
	5.40	95.5	9.5	12.9
Tricosanoic	0.09	99.9	12.0	13.2
acid	0.90	82.4	3.2	9.0
	5.40	89.0	5.1	13.4

### 2.5 实际样品测定

将建立的方法应用于4只健康的雄性SD大鼠和4只PFOS暴露的雄性SD大鼠肝脏中脂肪酸的检测。PFOS具有肝毒性^[11]^,本研究中SD大鼠在经口暴露PFOS后,血液甘油三酯和总胆固醇等肝功指标和肝脏病理切片均出现异常。每只大鼠肝脏中均能定量测得26种脂肪酸。经单因素方差分析,两组大鼠肝脏中共有11种脂肪酸出现显著性差异,其中PFOS暴露大鼠肝脏中C16∶0、C21∶0、C20∶3n6的含量出现显著性升高(*P*<0.05),其余8种脂肪酸含量则显著下降(*P*<0.05),详见表3。肝脏中脂肪酸等脂质的失调是肝炎、肝癌的危险因素^[15]^,其中C16∶0与其代谢物棕榈油酸(C16∶1)的比值(C16∶0/C16∶1)的增加与肝组织炎症程度相关^[3]^。PFOS暴露大鼠的C16∶0/C16∶1显著高于健康大鼠(*P*<0.05)。目前PFOS肝脏、神经等毒性的机制尚不明确,脂肪酸的种类和含量变化有可能作为其毒理机制研究的潜在指标之一。

**表3:** 健康大鼠和全氟辛烷磺酸暴露大鼠肝脏中差异脂肪酸的含量(*n*=4)

Compound	PFOS/(μg/mg)	Healthy/(μg/mg)
C12∶0	0.017±0.001	0.042±0.017
C16∶0	25.237±2.634	18.090±5.037
C20∶0	0.043±0.017	0.152±0.017
C20∶1	0.077±0.003	0.223±0.098
C21∶0	0.211±0.036	0.103±0.037
C20∶2	0.139±0.057	0.277±0.083
C22∶0	0.048±0.040	0.180±0.058
C20∶3n6	1.101±0.309	0.384±0.083
C20∶3n3	0.039±0.022	0.166±0.015
C24∶0	0.183±0.034	0.274±0.049
C20∶5	0.167±0.059	0.369±0.150

### 2.6 与其他方法的比较

将本研究所建立的方法与文献方法^[3,9,14,16]^进行对比(见表4)。文献方法至少需要5 mg及以上的肝脏样本量,能检测出13~24种脂肪酸。本方法仅使用1.1 mg肝脏即可检出39种脂肪酸,其中C15∶0、C18∶3n6和C18∶1n9t为其他文献中未检出的脂肪酸。此外,本方法还分离了文献方法未能分离的两对同分异构体脂肪酸C18∶2n6t和C18∶2n6c及C18∶1n9t和C18∶1n9c。不同的脂肪酸在维持健康或者疾病发展中发挥各自不同的作用,尽可能多地发现和分离更多的脂肪酸,对于发现潜在的疾病生物标志物或致病机理十分重要。

**表4:** 本方法与文献方法的对比

Reference	Sample amount of liver	Number of fatty acids	Separated isomers
This work	1.1 mg	39	C18∶2n6t, C18∶2n6c;
			C18∶1n9t, C18∶1n9c
[3]	not mentioned	13	none
[9]	100 mg	24	none
[14]	200 mg	23	none
[16]	5 mg	15	none

## 3 结论

本研究通过对样本前处理条件和色谱分离条件进行优化,建立了测定肝脏中39种脂肪酸组成和含量的GC-MS方法,并将其应用于健康和PFOS暴露SD大鼠肝脏中脂肪酸的检测。该方法样品处理步骤相对较简便,样本用量少,能检测的脂肪酸种类较多,且能有效分离同分异构体,为肝脏中脂肪酸的检测及研究提供了很好的方法,也为其他组织中脂肪酸的检测提供了参考方法。
